# Atomistic Insight on Effect of Silica Fume on Intermolecular Interactions between Poly(carboxylate) Superplasticizer and Calcium Ions in Concrete

**DOI:** 10.3390/nano14131084

**Published:** 2024-06-25

**Authors:** Berik Rakhimbayev, Bulat Mukashev, Parasat Kusherova, Abay Serikkanov, Ainagul Kemelbekova, Kamil Agybayev, Anuar Aldongarov, Nurlan Almas

**Affiliations:** 1 Institute of Physics and Technology, Satbayev University, Almaty 050013, Kazakhstan; b.rakhimbayev@sci.kz (B.R.); b.mukashev@sci.kz (B.M.); p.kusherova@sci.kz (P.K.); a.serikkanov@gmail.com (A.S.); a.kemelbekova@satbayev.university (A.K.); agybaevkamil@gmail.com (K.A.); 2 Department of Technical Physics, L. N. Gumilyov Eurasian National University, Astana 010000, Kazakhstan; enu-2010@yandex.kz; 3 Institute of Hydrogen Energy, International Science Complex Astana, Astana 010000, Kazakhstan

**Keywords:** poly(carboxylate), silica fume, concrete, atomistic models, binding

## Abstract

Understanding how poly(carboxylate)s of chemical admixtures interact with calcium ions in cement pore solutions in the presence of silica fume is fundamental to developing better chemical admixtures for concrete production. In this work, the intermolecular interactions of calcium ions with a poly(carboxylate) superplasticizer type of chemical admixture was investigated via classical all-atom molecular dynamics (MD) simulations and Density Functional Theory (DFT) calculation methods in the presence of silica fume. The classical all-atom MD simulation and DFT calculation results indicate that calcium ions are interacting with oxygen atoms of the carboxylate group of PCE. The better interaction energy could mean an improved adsorption of the PCE segment with calcium ions. In this regard, it can be noted that the ester-based PCE segment could have a better adsorption onto calcium ions in comparison with the ether-based PCE segment. Moreover, the presence of silicon dioxide could improve the adsorption of the PCE segment onto calcium ions.

## 1. Introduction

Concrete, a widely used construction material, primarily consists of aggregates such as sand and crushed stone, with cement acting as a binder, which is produced by heating a mixture of limestone (CaCO_3_) and other compounds [1]. However, cement production contributes to around 5–7% of global CO_2_ emissions, making it a significant source of greenhouse gases [2,3,4]. To address this issue, cement can be partially replaced with materials such as fly ash, silica fume, natural pozzolans, slag, calcinated clay, and concrete admixtures [1,2,3,4,5]. These additives, including silica fume, limestone, and fly ash, enhance the stability and workability of concrete, and improve its durability and compressive strength, but do not eliminate its tendency to fail in a brittle manner [5,6,7,8]. The selection and combination of these materials in the concrete mix are crucial due to their high fluidity and the need to reduce viscosity, which can be achieved by using chemical admixtures like superplasticizers and mineral admixtures like silica fume. Numerous researchers have attested that the use of both chemical and mineral admixtures results in superior properties for fresh and hardened concrete [9,10,11,12,13]. Superplasticizers, as chemical admixtures, have become essential in concrete production as they significantly improve the mechanical properties and reduce cement usage [13]. For instance, poly(carboxylate)s (PCEs) are a common type of chemical superplasticizer, characterized by comb-shaped copolymer polyelectrolytes with a linear backbone, mainly aliphatic, decorated with anionic groups like carboxylate, sulfate, sulfonate, phosphate, or phosphonate, and charge-neutral side chains containing oxyethylene repeating units [14,15]. The adsorption properties of these superplasticizers in cement depend on their performance in the presence of silica fume, an aqueous environment, and various ions (e.g., potassium, sodium, hydroxide, sulfate) under alkaline pHs. The conformational properties of superplasticizers are significantly influenced by calcium ions, which interact with the negatively charged groups of the superplasticizers [16].

### 1.1. Literature Review on the Application of Silica Fume in the Concrete Industry

While numerous studies have investigated the use of supplementary cementitious materials (SCMs) like fly ash, silica fume, and various superplasticizers in enhancing the properties of concrete, gaps remain in our understanding of their interactions at the molecular level. Soukal et al. (2020) examined the impact of different commercial poly(carboxylate) superplasticizers and silica fume on reactive powder concrete, highlighting significant variations in workability, specific weight, and mechanical properties depending on the superplasticizer used [17]. Their findings emphasize the importance of optimizing the superplasticizer and silica fume contents to achieve the best concrete properties. Hamada et al. (2021) discussed the environmental benefits of reusing silica fume in high-strength concrete, noting its positive influence on the mechanical properties and pore structure, despite potential drawbacks like reduced workability [18]. Anish et al. (2022) explored the synergistic effects of fly ash and silica fume in concrete, demonstrating improvements in strength and durability, especially when combined with superplasticizers [19]. Smarzewski (2019) reported that condensed silica fume could enhance the mechanical properties of high-performance concrete, particularly tensile strength and fracture energy, though the optimal replacement levels should not exceed 20% [20]. Lastly, Khan et al. (2022) investigated binary cementitious systems incorporating silica fume and micro-quartz filler, finding that these materials significantly reduce porosity and permeability while enhancing compressive strength [21]. These studies underscore the complex interactions between SCMs and superplasticizers, necessitating detailed molecular-level investigations to fully understand their synergistic effects in concrete mixtures [22,23]. This research aimed to fill this knowledge gap by employing advanced computational modeling techniques to elucidate the interactions between poly(carboxylate) superplasticizers, silica fume, and cement ions, thereby providing insights for optimizing concrete formulations for improved performance and sustainability.

This study hypothesized that the interactions between poly(carboxylate) superplasticizers and calcium ions in the presence of silica fume significantly influence the microstructural and mechanical properties of the concrete. Moreover, this study addressed the critical challenge of optimizing concrete formulations by investigating the molecular interactions between poly(carboxylate) superplasticizers and calcium ions in the presence of silica fume. Despite extensive research on concrete admixtures, a significant gap remains in understanding these interactions at the molecular level, which is essential for enhancing concrete’s performance and sustainability. The novelty of our research lies in employing advanced computational techniques, such as classical all-atom MD simulations and DFT calculations, to provide unprecedented insights into these interactions. By elucidating the synergistic effects of silica fume and chemical admixtures, our work paves the way for developing innovative admixtures that improve concrete’s durability, mechanical properties, and environmental impact.

### 1.2. Molecular Modeling Application for the Current Research

Despite the importance of understanding the interaction between different superplasticizers and cement ions like calcium, sodium, and potassium in the presence of silica fume, there is still limited knowledge at the molecular level. Computational modeling and simulations, such as coarse-grained molecular dynamics (MD), classical all-atom MD, and Density Functional Theory (DFT), have become crucial tools for exploring the microscopic properties of polyelectrolytes in concrete systems. In this context, Mohamed et al. reviewed computational models and simulations related to chemical admixtures in concrete systems, but the classical all-atom MD simulations have not yet been widely implemented to understand the intermolecular interaction of calcium ions with poly(carboxylate)-type concrete admixtures in the presence of silica fume [17].

To address this gap, we conducted classical all-atom MD simulations using the Gromos force field and DFT calculations with the B3LYP functional to estimate the interaction energies for the binding of calcium ions to poly(carboxylate)-type concrete chemical admixtures in the presence of silica fume. The theoretical model selected for the simulations was poly(carboxylate). In the following sections, the paper describes the methodology used for classical all-atom MD simulations and Density Functional Theory (DFT) calculations, along with snapshots, radial distribution functions, interaction energies, optimized structures, bond lengths, and HOMO–LUMO orbitals to investigate the intermolecular interactions of calcium ions with a poly(carboxylate)-type concrete chemical admixture in water and silica fume.

## 2. Materials and Methods

### 2.1. Theoretical Models

**Figure 1:**
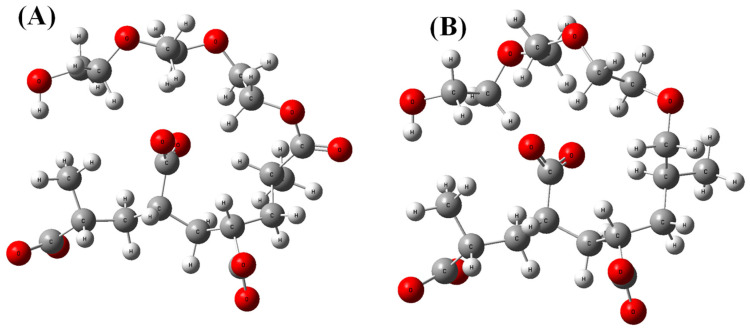
(**A**) Ester-based and (**B**) ether-based theoretical models of PCE superplasticizer segments. Color legend: white—hydrogen; grey—carbon; red—oxygen.

Silicon dioxide (SiO_2_) was selected as a theoretical model for silica fume, while calcium ions with chloride counter ions were selected as a representative theoretical model for cement minerals for our classical all-atom MD simulations. The two different PCEs’ structures including ester-based and ether-based monomers, as illustrated in Figure 1, were used to create a computational model of PCE superplasticizers for our classical all-atom MD simulations.

After the selection of theoretical models, we decided to create a classical all-atom MD simulation box with using one PCE segment with two calcium ions in the presence of chloride counter ions, 500 water molecules, and in the presence and absence of 5 silicon dioxide molecules.

### 2.2. Classical All-Atom Molecular Dynamics Simulations

Silicon dioxide was chosen as a theoretical representation of silica fume, while calcium ions in water were used as representative models of cement minerals. Additionally, Figure 1 illustrates representative segment models of PCE superplasticizers. The force field parameters (including dihedrals, angles, bonds, and partial charges) were optimized using the ATB database for two different PCE segments, as shown in Figure 1 [18]. Lennard–Jones (LJ) parameters were adopted from the standard GROMOS force field [19]. For the molecular dynamics (MD) simulations, a single representative segment with two calcium ions, one chloride counter ion, and 500 water molecules was designed in the presence and absence of 1/5/10 silicon dioxide molecules.

To prepare for the MD simulations, an initial simulation box containing the desired systems was created. Energy minimization using the steepest descent method was performed with a maximum force limit of 500 kJ/mol/nm on any atom to optimize the initial configuration at 298 K and 1 bar pressure for 1 ns. Subsequently, both NVT and NPT equilibrations were carried out at 298 K and 1 bar pressure for 1 ns each. Herein, we employed the NVT ensemble to stabilize the system’s temperature, also known as the “isothermal-isochoric” or “canonical” ensemble. The duration of this process varied depending on the system’s composition, whose aim is to have the temperature to reach a steady state at the desired value. If stabilization is not achieved, we extended the simulation time accordingly. Next, following temperature stabilization, we started the NPT ensemble to stabilize the system’s pressure and consequently its density. This equilibration of pressure occurred under the “isothermal–isobaric” ensemble, where the particle number, pressure, and temperature remained constant throughout the simulation. This sequential approach ensures that the system reaches equilibrium states for both temperature and pressure, which is essential for accurate and reliable molecular dynamics simulations. Once the system reached equilibrium, all-atom MD simulations were conducted for 10 ns with a temperature of 298 K and pressure of 1 bar using the NVT ensemble. During the simulation, the LINCS constraint algorithm was applied to all bonds. For the MD simulations, LJ and coulombic short-range interactions were cut off at 1.0 nm. Long-range interactions were computed using the Particle Mesh Ewald summation with a 0.16 nm grid spacing and fourth-order interpolation [20,21]. The V-rescale method was utilized to maintain the temperature, and the Berendsen pressure coupling method was implemented to maintain system pressure. Periodic boundary conditions were applied in all directions [22,23].

**Figure 2:**
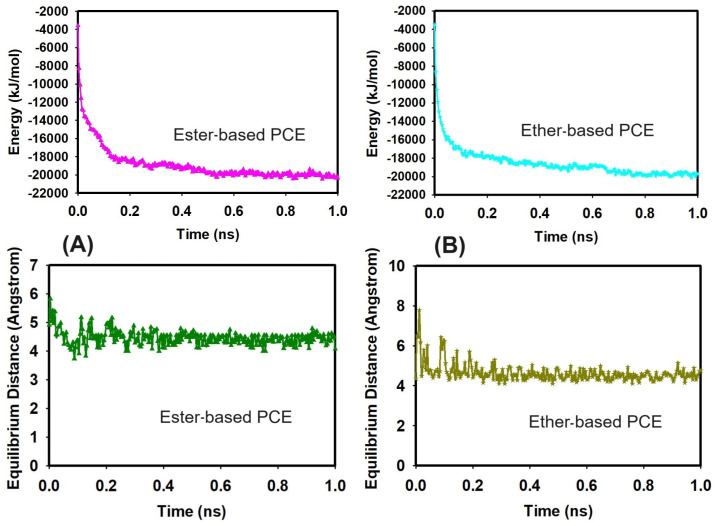
Graph of energy and equilibrium distance over time for (**A**) ester-based PCE and (**B**) ether-based PCE segments in the presence of Ca^2+^ ions and SiO_2_.

**Figure 3:**
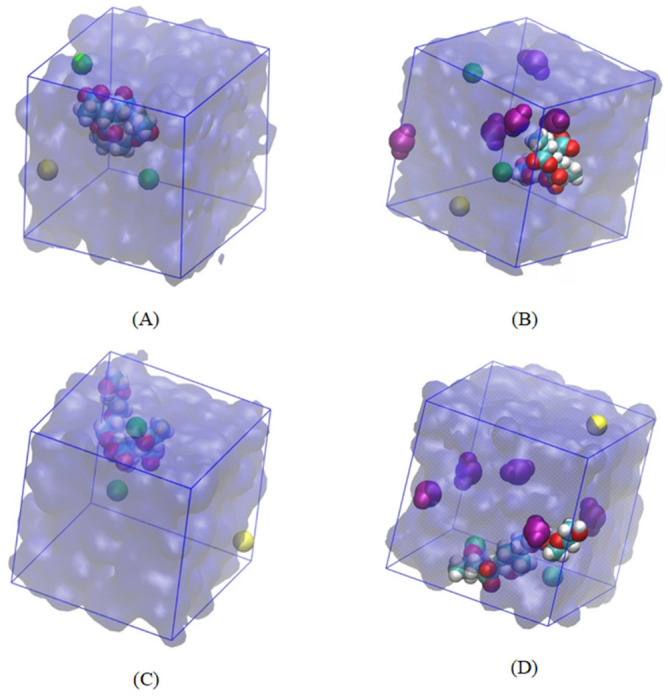
Simulation box of (**A**) ester-based PCE segment with calcium ions, (**B**) ester-based PCE segment with calcium ions in the presence of silicon dioxide, (**C**) ether-based PCE segment with calcium ions, (**D**) ether-based PCE segment with calcium ions in the presence of silicon dioxide. Color scheme: Water—quick surface, blue, transparent; silicon dioxide—VDM, purple; calcium ion—VDM, green; chloride ion—VDM, yellow; PCE—VDM, carbon is cyan, hydrogen is grey, and oxygen is red.

**Figure 4:**
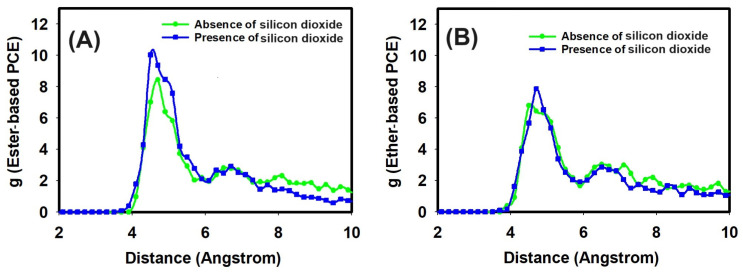
The radial distribution function of the interaction of calcium ion with (**A**) ester-based PCE segment and (**B**) ether-based PCE segment and water in the presence and absence of silicon dioxide.

**Figure 5:**
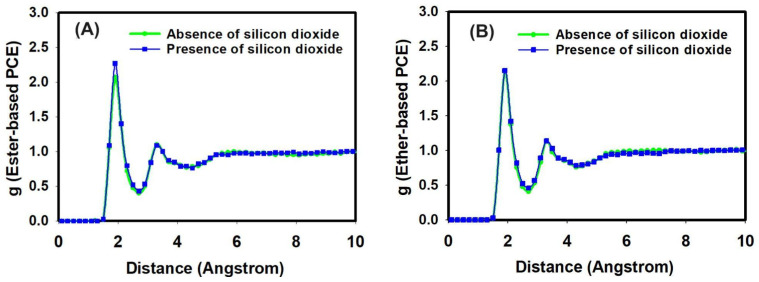
The radial distribution function of the interaction of water molecules with (**A**) ester-based PCE segment and (**B**) ether-based PCE segment in the presence and absence of silicon dioxide.

Regarding the output analysis of the classical all-atom MD simulations, energy and equilibrium distance, snapshots of MD simulations, the radial distribution functions, and the number of hydrogen bonds were calculated. We plotted the energy versus time (ns) and equilibrium distance over time (ns) after the equilibration phase to monitor the stability of our system (Figure 2). These graphs illustrate the stability and interaction dynamics of the ester- and ether-based poly(carboxylate) (PCE) segments in the presence of Ca^2+^ ions and SiO_2_. Secondly, we present snapshots from the production run of our all-atom classical MD simulations (Figure 3). These images display the ester- and ether-based PCE segments interacting with calcium ions, with and without the presence of silicon dioxide. Thirdly, the radial distribution functions (RDFs) were calculated from the production run of our classical all-atom MD simulations. Figure 4 focuses on the interaction of calcium ions with the ester- and ether-based PCE segments, while Figure 5 examines the interaction of water molecules with these PCE segments, both in the presence and absence of silicon dioxide. Lastly, Table 1 lists the number of hydrogen bonds formed between various PCE segments, water, and silicon dioxide calculated from the production run of our classical all-atom MD simulations.

The parameterization for the structures in our classical all-atom MD simulations was obtained using the GROMOS force field, with parameters generated from the ATB database. Calibration and verification of the force field parameters were performed by assessing the density of the system, which was approximately 1 g/cm^3^ at 298.15 K and 1 bar, indicating good agreement with the experimental data and a standard deviation of less than 2%. The validation of the force field was corroborated by existing literature [24,25,26], which supports the reliability of these parameters for similar systems. A sensitivity analysis was conducted by varying the composition of the designed system in the simulations, confirming the robustness of the interaction models under different conditions.

The simulations were performed using the GROMACS software (https://www.gromacs.org/), and the Visualization Molecular Dynamics (VMD) tool was used to visualize the simulated box and output analysis [27,28].

### 2.3. Density Functional Theory Calculations

**Figure 6:**
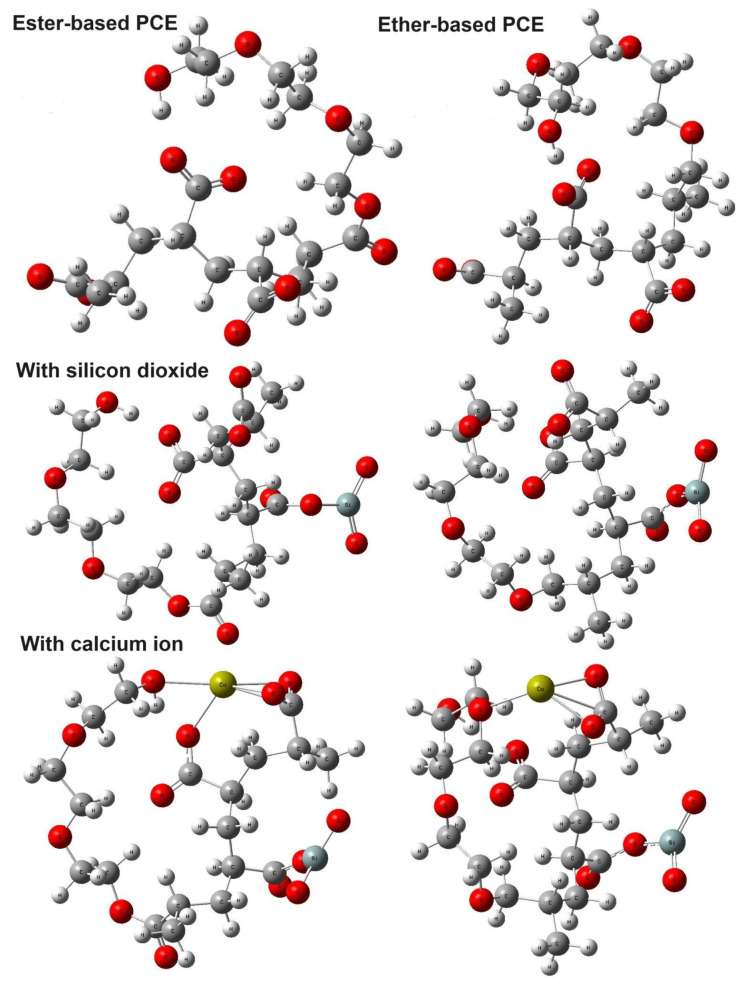
Optimized structures of ester-based PCE, ether based PCE, ester-based PCE + SiO_2_, ether-based PCE+ SiO_2_, ester-based PCE + SiO_2_ + Ca^2+^, and ether-based PCE+ SiO_2_ + Ca^2+^. White-gray atoms—hydrogen; red atoms—oxygen; grey atoms—carbon; blue-grey atoms—silicon; yellow atoms—calcium.

We also implemented DFT calculations for the ester- and ether-based PCE segments to estimate their binding energies with Ca^2+^ ions in the presence of SiO_2_. We used the standard B3LYP [29,30] functional in combination with 6-311+ G(d,p) basis sets implemented in Gaussian09 Rev. E.01 [31]. Visualization of the calculated DFT results was performed using the GaussView 5.0 program package. In the first step, we obtained optimized structures of the ester- and ether-based PCE monomers; then, using these structures, we calculated the structures of their complex with Ca^2+^ ions in the presence and absence of SiO_2_. The charge of the ester- and ether-based PCE monomers was −3 in our model. Figure 6 shows all the optimized structures. An analysis of the frequency calculations demonstrated the absence of negative frequencies for all structures, indicating that the obtained structures correspond to the global energy minimum.

Regarding the output analysis of the DFT calculations, snapshots of the optimized energy, bond length between the calcium ion and the oxygen atom of the carboxylate group of PCE, interaction energy, and HOMO–LUMO orbitals were calculated. In this regard, Figure 6 shows the optimized structures of the ester- and ether-based PCE segments with and without silicon dioxide and calcium ions. These structures were obtained using Density Functional Theory (DFT) calculations. The color scheme for the atoms is as follows: hydrogen, white; carbon, grey; oxygen, red; silicon, blue-grey; and calcium, yellow. Next, Table 2 presents the bond lengths between the oxygen atoms of the carboxylate groups in the ester- and ether-based PCE segments and the Ca^2+^ ions. These values were obtained from the closest bond lengths identified in our DFT optimized structures. Thirdly, Table 3 shows the interaction energies between Ca^2+^ ions and the ester/ether-based PCE segments in the presence of SiO_2_. The interaction energies were calculated.

The intermolecular interaction energy for the intermolecular interaction between a PCE monomer and calcium in the presence of SiO_2_ is described in (1).

(1) $$E_{int}=E(\mathrm{PCE}/{\mathrm{SiO}}_{2}/\mathrm{Ca}2^{+})-[E(\mathrm{PCE})+E({\mathrm{SiO}}_{2})+E(\mathrm{Ca}2^{+})]-\delta \left(BSSE\right)$$

where *E*(PCE/SiO_2_/Ca^2+^), *E*(PCE), *E*(SiO_2_), and *E*(Ca^2+^) are the total energies of the optimized PCE/SiO_2_/Ca^2+^ complex, and the pure/isolated PCE, SiO_2_, and Ca^2+^ molecules. The $\delta \left(BSSE\right)$ term corresponds to the corrections on the BSSE (Basis Set Superposition Error) using the counterpoise method for the pairs PCE/SiO_2_, PCE/ Ca^2+^, and SiO_2_/Ca^2+^.

**Figure 7:**
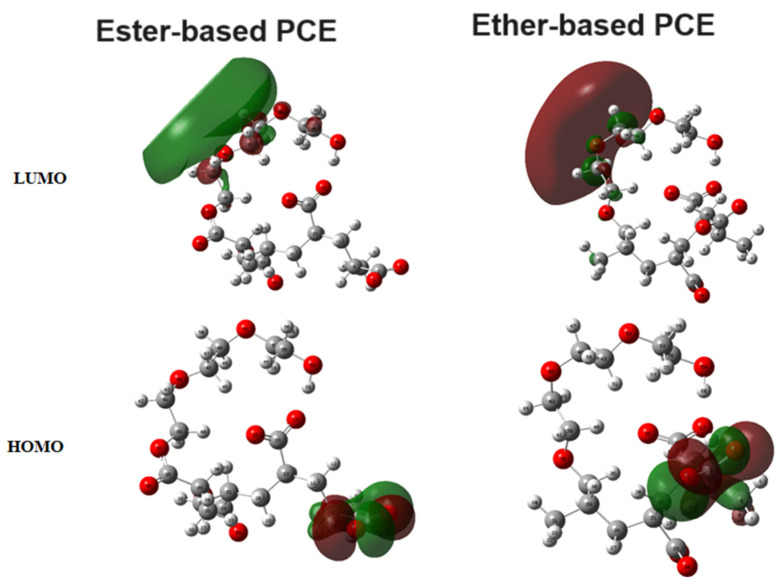
HOMOs and LUMOs of ester- and ether-based PCE monomers.

Lastly, the Highest Occupied Molecular Orbital (HOMO) and Lowest Unoccupied Molecular Orbital (LUMO) (Figure 7) for the ester- and ether-based PCE monomers were calculated from our DFT studies.

For our DFT calculations, we employed the B3LYP functional, which has been validated in the literature as accurate for estimating binding energies in systems similar to ours [29,30]. The results obtained from the DFT calculations were verified based on their consistency with the outcomes from classical all-atom MD simulations, providing a cross-validation between computational methods. In future work, we plan to conduct experimental validation to map these computational results.

## 3. Results and Discussion

### 3.1. Validation of the Stability of the System before Classical All-Atom MD Production Run

In this part, we double-checked the stability of the system before the classical all-atom MD production run. Namely, we checked the stability of the ester/ether-based PCE+Ca^2+^+SiO_2_ system over time. The graph of energy versus simulation time was plotted and can be seen in Figure 2.

In addition, the equilibrium distance between oxygen atoms of PCE and Ca^2+^ ions was around 4.5 Angstroms on average, with smaller fluctuations indicating that the designed system is stable over time in the presence of a water environment.

### 3.2. Classical All-Atom MD Simulation Results

The configurations of the PCE segments alongside water and calcium ions in the presence and absence of silicon dioxide are presented in Figure 3. In detail, the ester-based PCE segment in the presence of water molecules, calcium ions, and chloride counter ions are illustrated in Figure 3A. The interaction of silicon dioxide, calcium ions, and chloride counter ions with the ester-based PCE segment is shown in Figure 3B. The ether-based PCE segment in the presence of water molecules, calcium ions, and chloride counter ions are illustrated in Figure 3C. The interaction of silicon dioxide, calcium ions, and chloride counter ions with the ester-based PCE segment is illustrated in Figure 3D.

It is noteworthy that the ester/ether-based PCE segments were enveloped by calcium ions and water molecules, organizing into clusters that facilitated the binding of PCE with calcium ions. Notably, an addition of silicon dioxide led to a better covering of calcium ion and silicon dioxides around the ester/ether-based PCE segments to improve the binding of the PCE segments with calcium ions. To this end, classical all-atom MD simulations were executed to derive the radial distribution function (RDF) in the absence and presence of silicon dioxide for the PCE segment and calcium ions.

The interaction of calcium ions with the PCE segments and water was also studied by all-atom molecular dynamic simulations in the presence and absence of silicon dioxide. After the system reached equilibrium, the production runs of two designed systems were performed for 2 ns and used to analyze the interaction of calcium ions with two different PCE segments and water in the presence and absence of silicon dioxide in terms of radial distribution functions, which are shown in Figure 4.

Radial distribution functions (RDFs) are useful for explaining the two-body correlations in their positions. The RDF curves of the carboxylate group of the PCE segment with calcium ions are illustrated in Figure 4. Obviously, the distances between the first peaks of all the curves were larger than 4 Å. It was noted that calcium ions interacted with the carboxylate group of the ester-based PCE segment during the all-atom MD simulations, which could be further evaluated via RDF. Hence, the RDF curve between the oxygen atom of the carboxylate group of the ester-based PCE segment and calcium ions had a peak height of 8.43 at a peak position of 4.7 Angstroms in the absence of silicon dioxide. In the presence of silicon dioxide, the RDF between the oxygen atom of the carboxylate group of the ester-based PCE segment and calcium ions had a peak height of 10.02 at a peak position of 4.5 Angstroms, as can be seen in Figure 4A.

Regarding the ether-based PCE segment, the RDF between the oxygen atom of the carboxylate group of the ether-based PCE segment and calcium ions reached a peak height of 6.79 at a peak position of 4.5 Angstroms in the absence of silicon dioxide. In the presence of silicon dioxide, the RDF between the oxygen atom of the carboxylate group of the ether-based PCE segment and calcium ions reached a peak height of 7.87 at a peak position of 4.7 Angstroms, as can be seen in Figure 4B.

It can be noted that the presence of silicon dioxide increased the interaction of calcium ions with the PCE segments. Moreover, the ester-based PCE segment had a stronger intermolecular interaction with calcium ions in comparison with the ether-based PCE segment. Hence, the intermolecular interaction of the PCE segment with water molecules was also studied using RDF analysis.

The RDF curves of the oxygen atom of the carboxylate group of the PCE segments with water molecules are illustrated in Figure 5. Figure 5A illustrates the RDF between the oxygen atom of the carboxylate group of the ester-based PCE segment and hydrogen atoms of water molecules, while Figure 4B illustrates the RDF between the oxygen atom of the carboxylate group of the ether-based PCE segment and hydrogen atoms of water molecules in the absence and presence of silicon dioxide molecules.

The RDF peak height for the interaction of the oxygen atom of the carboxylate group of the ester-based PCE with hydrogen atoms of water molecules was 2.06 at a peak position of 1.90 Angstroms in the absence of silicon dioxide molecules. The presence of silicon dioxide yielded a similar peak height for the interaction of the oxygen atom of the carboxylate group of the ester-based PCE with hydrogen atoms of water molecules at around 2.27 at a peak position of 1.90 Angstroms, as can be seen in Figure 5A.

Regarding the ether-based PCE segment, the RDF between the oxygen atom of the carboxylate group of the ether-based PCE segment and water molecules reached a peak height of 2.14 at a peak position of 1.90 Angstroms in the absence of silicon dioxide. In the presence of silicon dioxide, the RDF between the oxygen atom of the carboxylate group of the ether-based PCE segment and water molecules reached a peak height of 2.15 at a peak position of 1.90 Angstroms, as can be seen in Figure 5B.

It can be noted that the presence of silicon dioxide did not change the interaction of water molecules with the PCE segments. Moreover, the ester-based PCE segment had a similar intermolecular interaction with water molecules as those of the ether-based PCE segment. Moreover, the initial pronounced peaks in the radial distribution function (RDF) for the oxygen atom within the carboxylate group of the PCE segment and the hydrogen atoms of the water molecules occurred at distances below 2 Å. These distances closely resemble the typical length of a conventional hydrogen bond, which is 1.8 Å. The formation of hydrogen bonds between the oxygen (O) atoms and the hydrogen (H) atoms validates the robust nature of hydrogen-bonded interactions for the PCE segments in their deprotonated state within highly alkaline solutions.

Hydrogen bonds assume a prevalent role within numerous chemical systems, thus serving as a pivotal factor in delineating intermolecular interactions among molecules. Consequently, the discourse surrounding the interaction of PCEs with water and silicon dioxide primarily hinges on the concept of hydrogen bonding, as elucidated in Table 1. To quantify the number of hydrogen bonds, this study applied geometric criteria, where the separation between the donor and acceptor was restricted to less than 0.35 nm, accompanied by an angle confinement within 30 degrees.

The outcomes, presented in Table 1, unveil the absence of hydrogen bonds between the PCE segments and silicon dioxide, for both the ester-based and ether-based PCE types, even in the presence of silicon dioxide. Conversely, a notable occurrence of hydrogen bonds manifested between the PCE segments and water, as well as between silicon dioxide and water within the systems. Interestingly, the introduction of silicon dioxide molecules did not induce any discernible alterations in the number of hydrogen bonds between the PCE segments and water molecules.

In summary, hydrogen bonds are indispensable in diverse chemical contexts, serving as a fundamental framework for understanding intermolecular associations. This study employed stringent geometric criteria to gauge the presence of hydrogen bonds, and while silicon dioxide appears to be devoid of hydrogen bonding with the PCE segments, noteworthy interactions emerged between the PCE segments and water, alongside interactions between silicon dioxide and water. Remarkably, the addition of silicon dioxide did not perturb the number of pre-existing hydrogen bonds between the PCE segments and water molecules.

In general, it was found that calcium ions interact with the oxygen atom of the carboxylate group of PCEs. The better interaction energy could mean improved an adsorption of the PCE segments on calcium ions. In this regard, it can be noted that the ester-based PCE segment could have a better adsorption onto calcium ion in comparison with the ether-based PCE segment. Moreover, the presence of silicon dioxide could improve the adsorption of PCE segments onto calcium ions.

### 3.3. DFT Calculation Results

As one can see from Figure 6, the Ca^2+^ ions form bonds with four oxygens of the ester-based PCE segment and with three oxygens of the ether-based PCE segment. The distances between oxygen atoms and Ca^2+^ ions are presented in Table 2. These data demonstrate that the bond lengths are shorter in the case of the ether-based PCE segment compared to that of the ester-based PCE segment but this was due to larger number of bonds for the ester-based PCE segment.

The analysis presented in Table 3 provides insights into the interaction energies and basis set superposition errors for the different systems. For the ester-based PCE + SiO_2_ + Ca^2+^ system, the calculated interaction energy was found to be −71.75 kJ/mol. On the other hand, the interaction energy for the ether-based PCE + SiO_2_ + Ca^2+^ system was determined to be −67.16 kJ/mol. These results indicate that the ester-based PCE system exhibits a slightly stronger interaction with SiO_2_ and Ca^2+^ ions compared to the ether-based PCE system. The negative values of the interaction energies signify attractive interactions between the components of each system.

Also, the HOMOs and LUMOs of the ester- and ether-based PCE monomers were obtained. Figure 7 demonstrates these molecular orbitals.

In Figure 7, the analysis reveals a notable localization of the Highest Occupied Molecular Orbitals (HOMOs) and Lowest Unoccupied Molecular Orbitals (LUMOs) in the ester- and ether-based (PCE) monomers. Specifically, the HOMOs in both monomers predominantly originated from the electrons associated with the oxygen atoms within the carboxylate groups. Conversely, the LUMOs exhibited strong localization on three adjacent carbon atoms in close proximity to the ether groups. This observation underscores the distinct molecular characteristics of these monomers, providing valuable insights into their electronic structure and reactivity. The localized nature of these molecular orbitals suggests a potential influence on the overall properties and performance of the PCEs, highlighting the significance of understanding these electronic features for optimizing their applications in various contexts.

### 3.4. Discussion of Classical All-Atom MD and DFT Calculation Results

This study’s findings align with those of recent extensive research on the effects of silica fume and superplasticizers on the properties of concrete. Previous studies emphasized the necessity of determining the optimal proportions of concrete mix components, including mineral and chemical admixtures. Such optimization can significantly reduce the cost, time, and number of experiments required for concrete production. The use of silica fume, known for improving the interfacial transition zone by decreasing porosity and increasing density, coupled with the workability enhancements provided by superplasticizers, underscores the synergy between these admixtures in improving both the fresh and hardened properties of concrete [29,30,31]. The literature further revealed that while superplasticizer inclusion generally enhances workability and early-age compressive strength. These insights reinforce our simulation results, which suggest that the interaction between poly(carboxylate) superplasticizers and calcium ions is enhanced by the presence of silica fume, potentially leading to improved adsorption and concrete properties [31,32,33]. Our findings provide a molecular-level understanding of these interactions, offering a foundation for optimizing admixture compositions in concrete production. Future experimental validation will be essential to corroborate these simulation results.

Moreover, the insights obtained from this study have substantial implications for the concrete admixture industry. By understanding the interactions between poly(carboxylate) chemical admixtures and calcium ions within cement pore solutions, especially in the presence of silica fume, we can significantly enhance the performance of superplasticizers [34,35,36,37]. The strong hydrogen-bonded interactions between the PCE segments and water molecules, along with the interaction with silicon dioxide, suggest that these materials can be engineered to improve the dispersion and stability of cementitious materials. The enhanced interaction energy of the ester-based PCE segments compared to the ether-based segments indicates potential for optimizing the composition of superplasticizers. This optimization can lead to better adsorption properties and improved workability and strength of concrete mixtures, ultimately resulting in more durable and reliable concrete structures [38,39]. The use of silica fume, a byproduct of the silicon and ferrosilicon industry, in conjunction with optimized PCEs, promotes the recycling of industrial waste, thus contributing to sustainability in the construction sector. This not only reduces the environmental footprint but also provides cost-effective solutions for enhancing concrete properties. 

Next, the findings from this study also have important implications for the field of wastewater technology. The molecular-level understanding of the interactions between PCEs and calcium ions can inform the development of advanced materials and treatment processes that improve the efficiency and sustainability of wastewater treatment systems. The strong hydrogen-bonded interactions and the superior adsorption properties of the ester-based PCE segments suggest that these materials can be tailored to achieve better performance in binding and removing contaminants from wastewater [40,41,42,43]. This is particularly important for the treatment of industrial effluents and other complex wastewater streams. Integrating silica fume with optimized PCEs in wastewater treatment processes can contribute to the development of eco-friendly technologies. The use of industrial byproducts not only helps in waste management but also improves the environmental sustainability of treatment systems.

## 4. Conclusions

In conclusion, this study delved into the essential realm of understanding the intricate interactions between poly(carboxylate) chemical admixtures and calcium ions within cement pore solutions, particularly in the presence of silica fume. By employing classical all-atom molecular dynamics (MD) simulations and Density Functional Theory (DFT) calculations, the intermolecular dynamics of calcium ions and poly(carboxylate) superplasticizers were rigorously explored, shedding light on the pivotal role of these interactions in concrete production.

The MD simulation and DFT calculation results provided compelling evidence that calcium ions establish significant interactions with the oxygen atoms within the carboxylate groups of poly(carboxylate) superplasticizers. Notably, the higher interaction energy observed hints at the potential for the enhanced adsorption of the poly(carboxylate) segments onto calcium ions. This finding becomes particularly pertinent in light of the distinction between ester-based and ether-based poly(carboxylate) segments, with the ester-based segments exhibiting superior adsorption capabilities onto calcium ions.

Furthermore, the presence of silicon dioxide, as represented by silica fume, emerges as a crucial factor augmenting the adsorption of poly(carboxylate) segments onto calcium ions. This suggests a synergistic effect between silica fume and the chemical admixtures, paving the way for improved concrete production processes.

This study’s findings confirm the working hypothesis that interactions between poly(carboxylate) superplasticizers and calcium ions, particularly in the presence of silica fume, significantly influence the microstructural and mechanical properties of concrete. The results from the MD simulations and DFT calculations support this hypothesis by demonstrating enhanced adsorption of poly(carboxylate) segments onto calcium ions, particularly with ester-based segments and in the presence of silica fume. These findings validate the hypothesized synergistic effects, highlighting the potential for optimizing concrete formulations through targeted molecular interactions.

In essence, this investigation contributes significantly to the field of concrete technology by unraveling the intricacies of the molecular interactions that govern the behavior of poly(carboxylate) chemical admixtures in the presence of calcium ions and silica fume. These insights lay the foundation for the development of advanced chemical admixtures tailored to optimize concrete properties, thereby advancing the realm of construction materials and facilitating the creation of more durable and sustainable concrete structures. Further exploration and application of these findings hold the promise of revolutionizing concrete production practices and positively impacting the construction industry as a whole.

## Figures and Tables

**Table 1:** Number of hydrogen bonds in the interactions of various PCE segments with water and silicon dioxide.

System Name		PCE Segment and Water	PCE Segment and SiO_2_	SiO_2_ and Water
Ester-based PCE segment	Absence of SiO_2_	19	-	-
	Presence of SiO_2_	20	0	7
Ether-based PCE segment	Absence of SiO_2_	19	-	-
	Presence of SiO_2_	19	0	6

**Table 2:** Bond lengths between oxygen atoms and Ca^2+^ ions in ester- and ether-based PCEs.

Compound	Bond Length in Å			
Ester-based PCE + SiO_2_ + Ca^2+^	Ca-O1	Ca-O3	Ca-O17	Ca-O58
	2.33	2.37	2.34	2.42
Ether-based PCE+ SiO_2_ + Ca^2+^	Ca-O1	Ca-O3	Ca-O52	-
	2.21	2.21	2.30	-

**Table 3:** Interaction energies between Ca^2+^ ions and ester/ether-based PCEs in the presence of SiO_2_.

Compound	Ester PCE + SiO_2_ + Ca^2+^	Ether PCE+ SiO_2_ + Ca^2+^
E(interaction), kJ/mol	−71.75	−67.16
$\delta \left(BSSE\right)$(PCE/SiO_2_), kJ/mol	5.91	9.09
$\delta \left(BSSE\right)$(PCE/Ca^2+^), kJ/mol	3.34	4.65
$\delta \left(BSSE\right)$(SiO_2_/Ca^2+^), kJ/mol	2.46	2.46

## Data Availability

The data presented in this article are available upon request from the corresponding author.
